# Effect of sulfate or hydroxychloride sources and levels of copper, manganese, and zinc on growth performance, carcass characteristics, and body weight variation of finishing pigs

**DOI:** 10.1093/tas/txag065

**Published:** 2026-05-16

**Authors:** Hilario M Cordoba, Mikayla S Spinler, Ethan B Stas, Rafe Q Royall, Jason C Woodworth, Robert D Goodband, Joel M DeRouchey, Mike D Tokach, Jordan T Gebhardt, Christiaan P A van de Ligt

**Affiliations:** Department of Animal Sciences and Industry, College of Agriculture, Kansas State University, Manhattan, KS, 66506-0201, United States; Department of Animal Sciences and Industry, College of Agriculture, Kansas State University, Manhattan, KS, 66506-0201, United States; Department of Animal Sciences and Industry, College of Agriculture, Kansas State University, Manhattan, KS, 66506-0201, United States; Department of Animal Sciences and Industry, College of Agriculture, Kansas State University, Manhattan, KS, 66506-0201, United States; Department of Animal Sciences and Industry, College of Agriculture, Kansas State University, Manhattan, KS, 66506-0201, United States; Department of Animal Sciences and Industry, College of Agriculture, Kansas State University, Manhattan, KS, 66506-0201, United States; Department of Animal Sciences and Industry, College of Agriculture, Kansas State University, Manhattan, KS, 66506-0201, United States; Department of Animal Sciences and Industry, College of Agriculture, Kansas State University, Manhattan, KS, 66506-0201, United States; Department of Diagnostic Medicine/Pathobiology, College of Veterinary Medicine, Kansas State University, Manhattan, KS, 66506-0201, United States; Selko USA, Indianapolis, IN, 46202, United States

**Keywords:** carcass characteristics, copper, finishing pig, growth, manganese, zinc

## Abstract

Two experiments were conducted to evaluate the effect of source and levels of Cu, Mn, and Zn on the performance, carcass characteristics, and body weight (BW) variability of finishing pigs. Experiment 1 used 2,025 pigs (337 × 1050, PIC; initially 39.9 ± 1.22 kg) with 27 pigs per pen and 15 pens per treatment. Treatments consisted of a control diet containing 30 mg/kg of Mn from MnSO_4_ (Eurochem, Veracruz, Mexico) or 15, 30, 45, or 65 mg/kg of Mn from Mn hydroxychloride (IBM; IntelliBond M, Selko, USA, LLC, Indianapolis, IN). From d 0 to 43, gain: feed ratio (G:F) increased (quadratic, *P* = 0.035) when Mn hydroxychloride increased up to 45 mg/kg but then decreased thereafter. There was no evidence for difference from increasing Mn hydroxychloride for any growth response in the overall period. When comparing Mn sources at 30 mg/kg of added Mn, there was no evidence of differences in any period or overall growth performance or carcass characteristics. In Exp.2, 1,026 pigs (337 × 1050, PIC; initially 25.9 ± 0.33 kg) were used with 27 pigs per pen and 19 pens per treatment. Treatments consisted of a control diet containing 110, 16, and 150 mg/kg of added Zn, Mn, and Cu, respectively, from sulfate sources or the same concentrations provided by hydroxychloride sources (IntelliBond, Selko USA, LLC, Indianapolis, IN). From d 0 to 61, there was a tendency (*P* = 0.052) for increased G:F when sulfate sources of Cu, Mn, and Zn were fed. From d 61 to 124, pigs fed hydroxychloride mineral sources increased (*P* = 0.041) ADG compared with those fed sulfate sources. However, there was no evidence for a difference in overall growth performance. A tendency (*P* = 0.054) for increased hot carcass weight (HCW) was observed in the final marketing event when pigs were fed hydroxychloride sources of Cu, Mn, and Zn, but no evidence of differences was observed in the first marketing or when all marketing data was combined. In both experiments, pigs were individually weighed to calculate within-pen BW coefficient of variation (CV), with no differences observed in BW variability. In conclusion, feeding hydroxychloride sources of Cu, Mn, and Zn resulted in no differences in overall growth performance, HCW, carcass yield, loin depth, backfat, and lean percentage compared with feeding sulfate sources; however, the G:F improvement when Mn hydroxychloride was increased up to 45 mg/kg and the tendency to increase HCW when feeding Cu, Mn, and Zn hydroxychloride warrants further investigation.

## Teaser

The hydroxychloride mineral sources used in this study did not affect growth performance of finishing pigs in comparison with sulfate sources of copper, manganese, and zinc.

## Introduction

Trace minerals are essential components of organs and tissues and play diverse roles in the body. They are involved in functions such as cell replication, signaling, and enzymatic reactions in carbohydrate, lipid, and protein metabolism (Suttle 2010). Manganese, a trace mineral necessary for reproduction and growth, is a component of superoxide dismutase and chondroitin sulfate synthesis. Zinc is involved in many enzymatic reactions as a component of metalloenzymes, cell signaling, and hormonal activity such as insulin. Copper is important for the synthesis of hemoglobin and red blood cells and activates several oxidative enzymes. It also promotes growth when feeding above requirement estimates (Espinosa 2019).

These minerals have relatively low bioavailability in feed ingredients typically used in swine diets (NRC 2012); therefore, trace mineral premixes are added to the diet. Due to their low cost, sulfate sources have been the preferred source to use for several years (Shurson et al. 2011).

Hydroxychloride trace minerals, such as Cu, Mn and Zn, are also inorganic sources recently introduced to the feed industry (Reddy et al. 2021). Hydroxychloride trace minerals differ from sulfate sources in that they possess a crystalline structure stabilized by covalent and coordination bonds, resulting in low water solubility and delayed dissociation (Villagómez Estrada et al. 2020). This delayed dissociation reduces premature interactions with dietary antagonists, such as phytate and other minerals, thereby maintaining higher mineral and phosphorus solubility in the digestive tract (Villagómez-Estrada et al. 2020, 2021). Importantly, sulfates do dissociate; however, their rapid ion release increases antagonistic reactions rather than improving absorption (Villagómez-Estrada et al. 2021).

According to NRC (2012), the requirement estimates for Cu, Mn, and Zn are 4 to 3 mg/kg, 2 mg/kg, and 60 to 50 mg/kg, respectively, from 25 to 135 kg of body weight (BW). A survey by Faccin et al. (2023) observed that the U.S. industry uses on average 15, 12, and 2 times more than the NRC (2012) requirement estimates of Cu, Mn, and Zn, respectively, in swine diets from 25 to 135 kg BW. The reason for feeding high concentrations of these trace minerals is to provide a margin of safety as many requirements studies date back at least 20 years ago (Oberleas et al. 1962; Dahmer et al. 1972; Apple et al. 2004). However, recent studies observed that feeding Zn and Cu above NRC (2012) requirement estimates improved growth performance and hot carcass weight (HCW) of finishing pigs (Coble et al. 2017; Cemin et al. 2019a; Espinosa 2019).

In both experiments, we hypothesized that different concentrations of Mn hydroxychloride, and the hydroxychloride sources of Cu, Mn, and Zn would provide improvements in growth performance and carcass characteristics compared to sulfate sources of these minerals due to differences in their solubility. To test our hypothesis, these studies evaluated the effects of increasing Mn from Mn hydroxychloride compared to a diet formulated to contain commercial concentrations of Mn from Mn sulfate. We also compared a feeding program using either sulfate or hydroxychloride mineral sources of Mn, Zn, and Cu on growth performance, carcass characteristics, and weight variation of finishing pigs housed in a commercial environment.

## Materials and methods

### General

The Kansas State University Institutional Animal Care and Use Committee approved the protocols (4847) used in these experiments.

### Experiment 1

A total of 2025 pigs (337 × 1050, PIC; initially 39.9 ± 1.22 kg) were used in a 95-d growth trial. The study was conducted at a commercial research-finishing site in southwest Minnesota (New Horizon Farms, Pipestone, MN). The barns were naturally ventilated and double-curtain-sided with totally slatted floors. Each pen (3.05 × 5.49 m) was equipped with a 5-hole stainless steel dry self-feeder (Thorp Equipment, Thorp, WI) and a bowl waterer for ad libitum access to feed and water. Pigs were housed in mixed gender pens with 27 pigs per pen and 15 pens per treatment. The treatments were structured as a completely randomized design and consisted of a control diet containing 30 mg/kg of Mn from MnSO_4_ (Eurochem, Veracruz, Mexico) or 15, 30, 45, or 65 mg/kg of Mn from Mn hydroxychloride (IBM; IntelliBond M, Selko USA, LLC, Indianapolis, IN). All treatment diets were manufactured at the New Horizon Farms Feed Mill in Pipestone, MN, and were formulated to meet or exceed NRC (2012) requirement estimates for growing-finishing pigs for their respective weight ranges (Tables 1 and 2). Daily feed additions to each pen were accomplished using a robotic feeding system (FeedPro; Feedlogic Corp., Wilmar, MN) able to record feed deliveries for individual pens. Diets were fed in meal form with phase 1 fed from 39.9 to 49.9 kg, phase 2 from 49.9 to 74.8 kg, phase 3 from 74.8 to 99.8 kg, and phase 4 from 99.8 to 133.3 kg. Experimental diets were corn-soybean meal-dried distiller grain with solubles (DDGS)-based and were formulated using a premix without Mn. All diets contained 150 mg/kg of Cu from IntelliBond C (Selko, Indianapolis, IN) and 100 mg/kg of Zn from IntelliBond Z (Selko, Indianapolis, IN). Manganese sources were added to the diet by hand-made premixes, which were added at the expense of corn.

**Table 1.** Composition of experimental diets, Exp. 1 (as-fed basis)^a^

Item	Phase 1	Phase 2	Phase 3	Phase 4
***Ingredient, %***				
*** Corn***	58.40	66.40	72.29	80.38
*** Soybean meal, 47.7% CP***	26.75	19.06	13.41	15.46
*** DDGS*** ^b^	10.00	10.00	10.00	–
*** Corn oil***	1.50	1.50	1.50	1.50
*** Limestone***	1.23	1.10	1.10	0.90
*** Monocalcium P, 21% P***	1.10	0.95	0.70	0.80
*** Salt***	0.35	0.35	0.35	0.35
*** L-Lys-HCl***	0.37	0.38	0.39	0.30
*** DL-Met***	0.06	0.03	0.01	0.04
*** L-Trp***	0.02	0.02	0.03	0.03
*** Thr*** ^c^	0.10	0.09	0.10	0.12
*** Vitamin-trace mineral premix*** ^d^ ^,^ ^e^	0.10	0.10	0.10	0.10
*** Phytase*** ^f^	0.02	0.02	0.02	0.02
*** Mn source*** ^g^	+/−	+/−	+/−	+/−
***TOTAL***	100	100	100	100
***Calculated analysis***				
***Standardized ileal digestible (SID) AA, %***				
*** Lys***	1.15	0.97	0.84	0.79
*** Ile: Lys***	63	61	60	61
*** Leu: Lys***	140	148	155	147
*** Met: Lys***	31	29	29	32
*** Met and Cys: Lys***	55	55	56	59
*** Thr: Lys***	62	62	64	65
*** Trp: Lys***	19	19	19	20
*** Val: Lys***	70	70	70	70
*** His: Lys***	42	42	42	43
***Total Lys, %***	1.31	1.11	0.96	0.89
***NE, kcal/kg***	2,499	2,550	2,586	2,605
***SID Lys: NE, g/Mcal***	4.62	3.82	3.25	3.04
***CP, %***	20.9	17.8	15.6	14.4
***Ca, %***	0.73	0.63	0.57	0.53
***STTP, %*** ^h^	0.52	0.47	0.41	0.39

a Phases 1, 2, 3, and 4 were fed from 39.9 to 49.9 kg, 49.9 to 74.8 kg, 74.8 to 99.8 kg, and 99.8 to 133.3 kg, respectively.

b DDGS, dried distiller grains with solubles.

c Thr Pro; CJ America-Bio, Downers Grove, IL.

d Provided per kg of diet: 110 mg Fe, 0.30 mg I, 0.30 mg Se, 1089 IU vitamin A, 272 IU vitamin D, 5.4 IU vitamin E, 1.2 mg vitamin K, 22.5 mg niacin, 7.5 mg pantothenic acid, 2.25 mg riboflavin, and 11 µg vitamin B12.

e Tribasic copper chloride (IntelliBond C, Selko, Indianapolis, IN) provided 150 mg/kg of Cu and Zinc hydroxychloride (IntelliBond C, Selko, Indianapolis, IN) provided 100 mg/kg of Zn.

f Optiphos Plus 2500 G (Huevepharma, Sofia, Bulgaria) was included at 1,500 FTU/kg providing an estimated release of 0.14% STTD P in all the feed phases.

g Mn hydroxychloride (IntelliBond M, Selko, Indianapolis, IN) or Mn sulfate (MnSO_4_, Eurochem, Veracruz, Mexico). The manganese source was added to the diet at the expense of corn.

h STTD P, standardized total tract digestible phosphorus.

**Table 2.** Composition of experimental diets, Exp. 2 (as-fed basis)^a^

Item	Phase 1	Phase 2	Phase 3	Phase 4
***Ingredient, %***				
*** Corn***	54.62	60.99	65.86	68.36
*** Soybean meal, 47.7% CP***	17.34	11.20	6.68	4.22
*** DDGS*** ^b^	25.00	25.00	25.00	25.00
*** Limestone***	1.45	1.40	1.30	1.25
*** Monocalcium P, 21% P***	0.30	0.20	−	−
*** Salt***	0.40	0.40	0.40	0.40
*** L-Lysine-HCl***	0.48	0.45	0.43	0.43
*** DL-Methionine***	0.02	−	−	−
*** L-Tryptophan***	0.03	0.03	0.03	0.04
*** Threonine*** ^c^	0.11	0.08	0.07	0.08
*** Vitamin-trace mineral premix*** ^d^	0.20	0.20	0.20	0.20
*** Phytase*** ^e^	0.05	0.05	0.03	0.02
***TOTAL***	100	100	100	100
***Calculated analysis***				
***Standardized ileal digestible (SID) AA, %***				
*** Lys***	1.08	0.91	0.78	0.72
*** Ile: Lys***	60	60	60	60
*** Leu: Lys***	155	168	183	190
*** Met: Lys***	29	30	33	34
*** Met and Cys: Lys***	56	59	64	66
*** Thr: Lys***	62	62	63	65
*** Trp: Lys***	18	18	18	18
*** Val: Lys***	71	73	76	77
*** His: Lys***	42	44	46	47
***Total Lys, %***	1.27	1.08	0.94	0.87
***NE, kcal/kg***	2,383	2,423	2,458	2,474
***SID Lys: NE, g/Mcal***	4.53	3.76	3.17	2.91
***CP, %***	20.4	17.9	16.1	15.2
***Ca, %***	0.67	0.62	0.53	0.50
***STTP, %*** ^f^	0.43	0.39	0.33	0.31

a Phases 1, 2, 3, and 4 were fed from approximately 25.9 to 49.9 kg, 49.9 to 74.8 kg, 74.8 to 99.8 kg, 99.8 to 137 kg, respectively.

b DDGS, dried distiller grains with solubles.

c Thr Pro; CJ America-Bio, Downers Grove, IL.

d Provided per kg of diet: 110 mg Fe, 0.30 mg I, 0.30 mg Se, 1,089 IU vitamin A, 272 IU vitamin D, 5.4 IU vitamin E, 1.2 mg vitamin K, 22.5 mg niacin, 7.5 mg pantothenic acid, 2.25 mg riboflavin, 11 µg vitamin B12 and 110 mg/kg of Zn, 16 mg/kg of Mn, and 150 mg/kg of Cu provided by sulfate sources or hydroxychloride sources (IntelliBond, Selko, Indianapolis, IN) were added to form the experimental treatments.

e Optiphos (Huevepharma, Sofia, Bulgaria) was included at 1,250 FTU/kg on phase 1 and 2, 625 FTU/kg on phase 3, and 500 FTU/kg on phase 4 providing an estimated release of 0.13% STTD P in phase 1 and 2, 0.11% STTD P in phase 3, and 0.10% STTD P in phase 4.

f STTD P, standardized total tract digestible phosphorus.

Pens of pigs were weighed approximately every 14 d to determine average daily gain (ADG), average daily feed intake (ADFI), and gain-to-feed ratio (G:F). On the last day of each trial, final pen weights were obtained, and pigs were tattooed with a pen identification number and transported to a U.S. Department of Agriculture-inspected packing plant (JBS Swift, Worthington, MN) for carcass data collection. Carcass measurements included HCW, loin depth, backfat depth, and percentage lean. Loin depth and backfat depth were measured by optical probe (SFK; Herlev, Denmark). Percentage lean was calculated from a plant proprietary equation. Carcass yield was calculated by dividing the pen average HCW by the pen average final live weight obtained at the farm.

On d 78, all pigs were individually weighed to determine BW variation. The same day, the 3 heaviest pigs in each pen were selected and marketed, corresponding to the first marketing event. These pigs were included in the calculation of pen growth performance, but not the carcass characteristics. On d 95, the remaining pigs were marketed, corresponding to the final marketing pigs, and included in the carcass characteristics evaluation.

### Experiment 2

A total 1026 pigs (337 × 1050, PIC; initially 25.9 ± 0.33 kg) were used in a 124-d growth trial. This study was conducted at the same commercial facility as Exp. 1; therefore, barn dimension, feed mill, feeding system, pen weights, feed data, and carcass data collection was conducted as in Exp.1. Pigs were housed in mixed gender pens with 27 pigs per pen and 19 pens per treatment. Treatments were structured as a completely randomized design and consisted of a control diet containing 110, 16, and 150 mg/kg of added Zn, Mn, and Cu, respectively, from sulfate sources or the same concentrations provided by hydroxychloride sources (IntelliBond, Selko USA, LLC, Indianapolis, IN). Diets were corn-soybean meal-DDGS-based and fed in meal form with phase 1 fed from 26 to 49.9 kg, phase 2 from 49.9 to 74.8 kg, phase 3 from 74.8 to 99.8 kg, and phase 4 from 99.8 to 136.8 kg.

On d 0 and 102, all the pigs in each pen were individually weighed to determine BW variation. On d 102, the 6 heaviest pigs in each pen were selected, tattooed with a pen identification number, and transported to the packing plant for carcass data collection, corresponding to the first marketing event. The remaining pigs were marketed on d 124, corresponding to the final marketing event pigs. For this experiment, carcass characteristics were collected at the first and final marketing event for evaluation of the individual events and overall. The individual HCW collected at the packing plant was analyzed to determine variation within and between treatments.

#### Chemical analysis

For both experiments, representative diet samples were collected from all the feeders of each treatment by phase and stored at −20°C until analysis. Samples of the diets were combined within the dietary treatment and composite samples per treatment and phase were sent to a commercial laboratory (Cumberland Valley Analytical Services, Waynesboro, PA) where they were analyzed for dry matter (method 935.29; Association of Official Agricultural Chemists (AOAC) International, 1990), crude protein (990.03, AOAC International 2006), calcium (Ca) and phosphorus (P; method 985.01; AOAC International 2006), Cu, Zn, and Mn (Method 985.01; AOAC International 2006).

#### Statistical analysis

Data from both studies were analyzed as a completely randomized design for one-way ANOVA using the lmer function from the lme4 package in R (version 4.1.1 (2021-08-10), R Foundation for Statistical Computing, Vienna, Austria) with pen considered the experimental unit, and treatment as fixed effect. For Experiment 1, contrast coefficients were used to evaluate linear and quadratic effects of Mn concentration within the hydroxychloride source and pairwise comparison of Mn sources at 30 mg/kg. In both experiments, pig individual weight data on d 78 (Exp. 1) and d 0 and 102 (Exp.2) were used to calculate within-pen coefficient of variation which was analyzed in a similar manner to growth data. Hot carcass weight was used as covariate for carcass characteristics analysis. All results were considered significant at *P* ≤ 0.05 and marginally significant between *P* > 0.05 and *P* ≤ 0.10.

## Results and discussion

### Chemical analysis

Considering the trace minerals from basal ingredients, analyzed dietary Cu, Mn, and Zn were consistent with calculated values used in diet formulation in both experiments (Tables 3 and 4).

**Table 3.** Chemical analysis of Exp. 1 diets (as fed-basis)^a^

	MnSO_4_	Mn Hydroxychloride			
***Calculated Mn, mg/kg***	30	15	30	45	65
***Analyzed Mn, mg/kg***	46	32	41	57	65

a Values represent means from 20 composite samples (4 samples per treatment). For each treatment, samples were collected from multiple feeders, blended, subsampled, ground, and analyzed (Cumberland Valley Analytical Services, Waynesboro, PA).

**Table 4.** Chemical analysis of Exp. 2 diets (as fed-basis)^a^

	Mineral source^b^	
	Sulfate	Hydroxychloride
***Calculated, mg/kg (added basis)***		
*** Zn***	110	110
*** Mn***	16	16
*** Cu***	150	150
***Analyzed, mg/kg (total basis)***		
*** Zn***	211	220
*** Mn***	30	36
*** Cu***	168	151

a Values represent means from 8 composite samples (4 samples per treatment). For each treatment, samples were collected from multiple feeders from the two groups, blended, subsampled, ground, and analyzed (Cumberland Valley Analytical Services, Waynesboro, PA).

b Sulfate or hydroxychloride sources of Zn, Mn, and Cu were used for the 2 treatments.

### Growth performance and carcass characteristics

In Exp.1, from d 0 to 43, G:F increased (quadratic, *P* = 0.035) with increasing Mn hydroxychloride up to 45 mg/kg but then decreased thereafter (Table 5). There was no evidence for difference from increasing Mn hydroxychloride for any growth response criteria from d 43 to 95 or d 0 to 95. When comparing Mn sources at 30 mg/kg added Mn, there was no evidence of differences  in any period or for overall growth performance. For carcass characteristics, no evidence of difference was observed for any criteria when comparing pigs fed increasing Mn hydroxychloride or Mn sulfate in diets at 30 mg/kg.

**Table 5.** Effect of increasing Mn hydroxychloride on finishing pig growth performance and carcass characteristics, Exp. 1^a^

	MnSO_4_^b^, mg/kg	Mn Hydroxychloride^c^, mg/kg					*P* =^d^		
Item	30	15	30	45	65	SEM	Source	Linear	Quadratic
***BW, kg***									
*** d 0***	39.9	39.9	39.9	39.9	39.9	1.22	0.988	0.977	0.994
*** d 43***	80.0	79.9	80.4	80.1	80.4	2.78	0.839	0.828	0.984
*** d 78***	109.1	109.5	110.1	109.2	110.8	2.56	0.597	0.626	0.721
*** d 95***	133.0	133.3	133.3	132.2	133.4	3.86	0.832	0.857	0.671
***d 0 to 43***									
*** ADG, kg***	0.93	0.93	0.93	0.93	0.94	0.042	0.949	0.468	0.940
*** ADFI, kg***	2.11	2.13	2.13	2.08	2.16	0.091	0.627	0.693	0.207
*** G:F, g/kg***	443	436	438	449	433	4.0	0.461	0.941	0.035
***d 43 to 95***									
*** ADG, kg***	1.04	1.03	1.04	1.02	1.02	0.018	0.901	0.430	0.676
*** ADFI, kg***	3.14	3.13	3.15	3.08	3.12	0.047	0.821	0.579	0.782
*** G:F, g/kg***	332	329	330	332	328	9.0	0.598	0.883	0.359
***d 0 to 95***									
*** ADG, kg***	0.99	0.98	0.99	0.98	0.98	0.013	0.930	0.876	0.689
*** ADFI, kg***	2.66	2.66	2.67	2.61	2.67	0.068	0.753	0.874	0.467
*** G:F, g/kg***	373	369	370	375	368	5.0	0.528	0.945	0.123
***Individual pig weight CV, %*** ^e^									
***d 78***	10.6	10.7	11.5	10.6	11.3	0.92	0.186	0.634	0.927
***Carcass characteristics***									
*** HCW, kg***	98.9	99.4	98.9	97.6	99.0	2.60	0.985	0.566	0.283
*** Carcass yield, %***	73.6	74.3	74.2	73.6	73.8	0.50	0.407	0.368	0.746
*** Backfat depth, mm*** ^f^	17.8	18.1	18.0	17.8	17.9	0.97	0.551	0.424	0.706
*** Loin depth, mm*** ^f^	64.7	65.1	65.1	65.8	65.4	1.28	0.514	0.411	0.654
*** Lean, %*** ^f^	55.8	55.6	55.7	55.9	55.8	0.45	0.764	0.284	0.605
***Mortality and removals, %***	3.21	6.17	5.68	3.46	6.67	6.441	0.746	0.955	0.739

a A total of 2,025 pigs (initial BW of 39.9 ± 1.22 kg) were used in two groups with 27 pigs per pen and 15 replicates per treatment.

b Erachem, Veracruz, Mexico.

c IntelliBond M (IBM), **Selko** USA, Indianapolis, IN.

d Contrast coefficients were used to compare the effect of manganese sources at 30 mg/kg level and linear and quadratic effect within the increasing levels of IntelliBond M.

e At the first marketing event, a total of 1,937 pigs were weighted individually to calculate variation of final BW. Coefficient of variation of individual pig weight was calculated for each pen and analyzed to determine the effect of dietary treatment with pen as the experimental unit.

f Adjusted using HCW as covariate.

Kerkaert et al. (2021) observed that feeding 8, 16, and 32 mg/kg of Mn from Mn sulfate or hydroxychloride to pigs from 70 to 130 kg BW resulted in no differences in growth performance. Some studies have observed increased or tendencies to increase G:F in the grower (35 to 70 kg) stage when utilizing inorganic and chelated sources of Mn at 24 to 88 mg/kg (Kats et al. 1994) and Mn amino acid complex sources at greater inclusions such as 350 to 700 mg/kg (Apple et al. 2004; Sawyer et al. 2007). Based on our findings and others, G:F appears to be improved in the early stages of finishing (40 to 80 kg) period with increasing inclusion of Mn, but not for the entire finishing phase. Studies using different sources and levels of added Mn did not observe evidence for differences when measuring hot carcass weight, carcass yield, and backfat (Kats et al. 1994; Sawyer et al. 2007; Schwarz et al. 2017). However, Kerkaert et al. (2021) observed a tendency for a quadratic increase in carcass yield when increasing added Mn from Mn hydroxychloride from 8 to 16 mg/kg, but a decrease thereafter. This response was not observed in our study as the lowest concentration was 15 mg/kg of added Mn.

In Exp. 2, from days 0 to 61, there was a tendency (*P* = 0.050) for increased G:F when sulfate sources of Cu, Mn, and Zn were fed while from d 61 to 124, pigs fed Cu, Mn, and Zn hydroxychloride sources had increased (*P* = 0.041) ADG (Table 6). However, there were no differences in growth performance in the overall (d 0 to 124) finishing period. No evidence of difference was observed for any carcass criteria when comparing pigs fed sulfate or hydroxychloride mineral sources at the first marketing event. However, there was a tendency (*P* = 0.054) for increased HCW in the final marketing event when pigs were fed hydroxychloride sources of minerals, although when both marketing events were combined, no evidence of differences (*P* > 0.10) was observed.

**Table 6.** Effect of sulfates and hydroxychloride mineral sources feed program on growth performance, weight distribution and carcass characteristics of finishing pigs, Exp. 2^a^

	Mineral source			
Item	Sulfates^b^	Hydroxychloride^c^	SEM	*P* =
***BW, kg***				
*** d 0***	25.9	26.0	0.33	0.865
*** d 61***	78.6	78.5	0.49	0.886
*** d 102***	116.0	116.7	0.59	0.405
*** d 124***	135.5	136.8	0.68	0.192
***d 0 to 61***				
*** ADG, kg***	0.858	0.855	0.0044	0.656
*** ADFI, kg***	1.98	2.01	0.0146	0.269
*** G:F, g/kg***	433	427	2.3	0.050
***d 61 to 124***				
*** ADG, kg***	0.935	0.960	0.0084	0.041
*** ADFI, kg***	2.98	3.02	0.022	0.188
*** G:F, g/kg***	314	318	2.6	0.295
***d 0 to 124***				
*** ADG, kg***	0.895	0.906	0.0046	0.115
*** ADFI, kg***	2.47	2.50	0.0161	0.203
*** G:F, g/kg***	363	363	2.1	0.963
***Individual pig weight CV, %*** ^d^				
*** d 0***	15.6	15.5	0.59	0.919
*** d 102***	9.8	9.2	0.37	0.226
***Carcass characteristics***				
*** First marketing event***				
*** HCW, kg***	92.8	92.8	0.73	0.948
*** HCW CV, %***	5.5	4.9	0.35	0.294
*** Carcass yield, %***	73.1	73.3	0.27	0.655
*** Backfat depth, mm.*** ^e^	16.18	16.09	0.312	0.842
*** Loin depth, mm.*** ^e^	60.79	60.61	0.613	0.830
*** Lean, %*** ^e^	56.3	56.4	0.22	0.818
*** Final marketing event***				
*** HCW, kg***	98.0	99.7	0.582	0.054
*** HCW CV, %***	8.2	7.7	0.35	0.291
*** Carcass yield, %***	72.1	72.4	0.26	0.467
*** Backfat depth, mm.*** ^e^	15.68	16.07	0.202	0.176
*** Loin depth, mm.*** ^e^	63.12	63.05	0.390	0.900
*** Lean, %*** ^e^	56.9	56.7	0.14	0.208
*** Overall***				
*** HCW, kg***	96.7	97.8	0.55	0.189
*** HCW CV, %***	7.5	6.9	0.28	0.170
*** Carcass yield, %***	72.4	72.7	0.01	0.353
*** Backfat depth, mm.*** ^e^	15.79	16.09	0.169	0.208
*** Loin depth, mm.*** ^e^	62.52	62.41	0.302	0.809
*** Lean, %*** ^e^	56.8	56.6	0.12	0.252
***Total removals, %***	3.7	5.3	0.99	0.226

a A total of 1,026 pigs (initial BW of 25.9 kg ± 0.33 kg) were used with 27 pigs per pen and 19 replicates per treatment. Treatments were assigned in a completely randomized design to compare the effect of sulfates and hydroxychloride (IntelliBond, Selko, Indianapolis, IN) sources on growth performance, weight distribution and carcass characteristics of finishing pigs.

b Cu, Mn, and Zn were provided by sulfate sources at 150, 16, and 110 mg/kg, respectively.

c Cu, Mn, and Zn were provided by hydroxychloride sources at 150, 16, and 110 mg/kg, respectively.

d Pigs were weighed individually to calculate variation of final BW. Coefficient of variation of individual pig weight was calculated for each pen and analyzed to determine the effect of dietary treatment with pen as the experimental unit.

e Adjusted using HCW as covariate.

Mendonça et al. (2021) observed improvements in ADG, individual BW, and HCW when feeding 150 and 80 mg/kg of added Cu and Zn hydroxychloride, respectively, compared to the same levels provided by CuSO4 and ZnO to pigs from 27.7 to 104 kg. Hernández et al. (2008) observed improvements in G:F when using proteinate amino acid chelate sources of Zn and Cu compared to sulfate sources from 25 to 107 kg, regardless of the inclusion level utilized. However, there was a tendency for G:F improvement when Cu was included at 156 mg/kg and Zn at 170 mg/kg. In addition, a meta-analysis of six different trials reported improvements in ADG and G:F when using 80 mg/kg of Zn from hydroxychloride sources compared to sulfate sources (Van Kuijk et al. 2019). Improvements in ADG, ADFI, G:F, final BW, and HCW were observed by Knauer and Van de Ligt (2022) when using 150 mg/kg of added Cu hydroxychloride to pigs from 30 to 123 kg BW in comparison with another hydroxychloride source of Cu. The results from the cited literature show responses on growth performance and carcass traits in some of the studies and at certain stages of growth. However, like in our study, most studies showed no benefit of using an alternative source compared to sulfates when supplementing minerals from 19 to 133 kg (Creech et al. 2004; Cemin et al. 2019b; Kerkaert et al. 2021), indicating that mineral supplementation is not a limiting factor for pig growth.

Mendonça et al. (2021) observed improvements in ADG, final BW, and HCW when using 150 mg/kg of added Cu hydroxychloride compared to the same levels provided by CuSO_4_. In addition, despite the lack of significance, Coble et al. (2017) observed 2.2 kg greater HCW when using 150 mg/kg of Cu hydroxychloride compared to the same level provided by CuSO_4_. This is similar to the improvement from hydroxychloride sources over the sulfate sources in ADG observed during the finisher period in our study and the tendency for heavier HCW at the final marketing event. However, like in our study, most studies showed no benefit of using an alternative source compared to sulfates when supplementing minerals from 19 to 133 kg (Creech et al. 2004; Cemin et al. 2019b; Kerkaert et al. 2021), indicating that mineral supplementation is not a limiting factor for pig growth.

### Body and hot carcass weight variation

There was no evidence of difference in the CV of pig BW between treatments at d 78 in Exp. 1 and on d 0 or d 102 of Exp. 2 (Tables 5 and 6). For HCW variability, no evidence of difference was observed when comparing sulfates and hydroxychloride mineral sources in pigs evaluated on the first, final, and combined marketing events.

Pigs have an inherent decrease in BW variability from birth to market when expressed as CV (Tolosa et al. 2021). In a meta-analysis of pig BW variation from birth to market, Tolosa et al. (2021) observed BW CV was approximately 21% and 10% at birth and marketing, respectively. Some authors studied reducing pigs’ BW CV by sorting pens by weight categories (O’Quinn et al. 2000; Brumm et al. 2002; Wolter et al. 2002; Hastad et al. 2020); however, we are not aware of any studies that evaluated mineral source or level on pig body weight variability. Our results demonstrate that using different levels and sources of Mn or sources of Cu, Mn, and Zn, did not affect the CV of pig BW.

## Conclusion

In conclusion, these results suggest that increasing levels of Mn hydroxychloride or using hydroxychloride sources of Cu, Mn, and Zn together in growing-finishing pig diets did not improve overall growth performance and carcass characteristics compared with those fed sulfate sources. Additionally, the tendency to increase HCW when feeding hydroxychloride sources of Cu, Mn, and Zn warrants further investigation.

## List of abbreviations

ADFI: average daily feed intake
ADG: average daily gain
BW: body weight
CV: coefficient of variation
DDGS: dried distiller grain with solubles
G: F: gain-to-feed ratio
HCW: hot carcass weight
NE: net energy
